# Editorial: Evolutionary genomics of *Candidatus* Liberibacter spp. and their interactions with plant and insect-vector hosts

**DOI:** 10.3389/fmicb.2022.1025795

**Published:** 2022-10-06

**Authors:** Xuefeng Wang, Kranthi Mandadi, Leandro Peña, Mengji Cao, Changyong Zhou

**Affiliations:** ^1^National Citrus Engineering Research Center, Citrus Research Institute, Southwest University, Chongqing, China; ^2^Department of Plant Pathology and Microbiology, Texas A&M University, College Station, TX, United States; ^3^Texas A&M AgriLife Research and Extension Center, Texas A&M University System, Weslaco, TX, United States; ^4^Institute for Advancing Health Through Agriculture, Texas A&M AgriLife, College Station, TX, United States; ^5^Laboratório de Biotecnologia Vegetal, Fundo de Defesa da Citricultura (Fundecitrus), Araraquara, São Paulo, Brazil; ^6^Instituto de Biología Molecular y Celular de Plantas, Consejo Superior de Investigaciones Científicas (IBMCP-CSIC), Valencia, Spain

**Keywords:** *Candidatus* Liberibacter spp., Huanglongbing, Zebra chip disease, interactions, omics, integrated pest management

Multiple pathogenic species of “*Candidatus* Liberibacter” have been identified from plant hosts worldwide. Among these species, “*Ca*. Liberibacter asiaticus” (*C*Las) and “*Ca*. Liberibacter solanacearum” (*C*Lso) are the putative causal agents of citrus Huanglongbing (HLB) and Zebra chip disease (ZC), respectively, which are highly destructive toward the citrus and potato industries. Due to their phloem-colonized nature, the lack of pure culture, and their intracellular life in plant hosts, studies on the biology and control of “*Ca*. Liberibacter” species are facing significant challenges. Although genomic information of the “*Ca*. Liberibacter” species has been accumulated rapidly and some virulence factors have been dissected, how the three-way interactions within pathogen-insect-plant occur remains further characterization.

The purpose of the Research Topic was to publish high-quality research papers and review articles focusing on the following: (1) comparative and functional genomics of “*Ca*. Liberibacter” spp.; (2) multi-omics of plant and insect-vector hosts in response to *C*Las/*C*Lso infection; (3) characterization of effectors or other virulence traits and their interactions with plant and insect-vector hosts; (4) development of genetic and genome-editing strategies against *C*Las/*C*Lso. A total of 18 manuscripts, including original research and reviews, have been received, of which 13 were eventually accepted, comprising two reviews and 11 research papers.

Currently, *Ca*. Liberibacter is only found in Psylloidea vector hosts but not in other insect superfamilies. To discover new *Liberibacter* species and determine their phylogenetic relationship, psyllid samples from 44 species of 35 genera of five plant families, collected from 11 representative geographical locations, were used for 16S rRNA sequencing. A novel “*Ca*. Liberibacter” species, “*Ca*. Liberibacter capsica,” was identified based on the phylogenomic analyses of sequencing data (Kwak et al.). Three types of phages/prophages (Type 1, Type 2, and Type 3) have been characterized in *C*Las according to the presence of circular plasmid sequences. It is generally believed that more *C*Las phages/prophages are likely to be found. A novel *Microviridae* phage, *C*LasMV1, with a small circular genome was identified in a Chinese *C*Las strain (GDHZ11) (Zhang et al.). *C*LasMV1 was frequently detected with a high copy number in a *C*Las population from southern China. Bacteriomic analyses of Asian citrus psyllids and citrus samples from California identified ten bacterial generas of endophytes, including *Bradyrhizobium, Buchnera, Burkholderia*, “*Ca*. Profftella armature,” and *Mesorhizobium*. *Bradyrhizobium* and *Mesorhizobium* DNA were identified as interfering with *C*Las detection using the conventional 16S rRNA gene-based PCR due to their sequence similarity, particularly at low or zero *C*Las titer situations, likely providing false positive results (Huang et al.).

Understanding the early events of the interaction between *C*Las-plant hosts may help to develop bacterial control strategies. To better understand *C*Las dynamics and whole-plant colonization during the earliest stage of infection, Alves et al. identified the key time course of *C*Las proliferation in *Citrus sinensis* “Valencia” (susceptible host), in *Murraya paniculata* (transient host), and in *Bergera koenigii* (resistant) under similar initial inoculum pressure. Fang et al. found a high level of *C*Las population in fruit pith tissue compared with other tissues. The high-resolution *C*Las transcriptome profiles were generated using the *C*Las-abundant fruit pith samples for dual RNA-seq, suggesting that fruit pith could be an ideal host material for further studies on *C*Las–citrus interaction. The non-natural host periwinkle (*Catharanthus roseus*) infected *via* dodder (*Cuscuta campestris*) from *C*Las-infected citrus plants was subjected to the analysis of transcriptomics and small-RNA profiling. miR164-NAC1, miR828-MYB94/miR1134-HSF4, and miR827-ATG8 regulatory networks were identified as critical miRNA–mRNA regulatory channels, showing new potential connections in periwinkle–*C*Las interactions (Zeng et al.). Currently, our knowledge of the cellular function of *C*Las secretory proteins in plant or insect hosts is limited. *C*aLasSDE115 effector was proposed as an invasion-associated locus B of *C*Las, participating in the early bacterial colonization of citrus. It could also regulate citrus resistance to HLB *via* modulating the transcriptional regulation of systemic acquired resistance (SAR)-related genes (Du et al.).

To understand the molecular interactions of *C*Las-*Diaphorina citri* during the vector acquisition process, cDNA libraries obtained from the gut of adult psyllids were sequenced on an Illumina platform. A high copy number of genes (up to 95%) were expressed in the initial step of *D. citri* gut colonization through read mapping of *C*Las genes, suggesting such genes might play critical roles in *C*Las colonizing insect organs (Darolt et al.). Tang et al. found that the expression of IAPP5.2 gene (the inhibitor of apoptosis) was significantly induced during the period that *C*Lso translocated into the gut cells. Detection of apoptosis level, *C*Lso acquisition, and transmission efficiency after silencing IAPP5.2 suggested that *C*Lso might repress the apoptotic response in the psyllid guts by upregulating IAPP5.2 at the early stages of *C*Lso infection.

Identifying novel genetic resistance and tolerance sources to *C*Las/*C*Lso can be valuable components for the integrated management of HLB/ZC. A taxonomically diverse collection of tuber-bearing *Solanum* species was screened, and a ZC-resistant accession (*Solanum berthaultii*) was identified among the 52 screened accessions. The differences in leaf trichome density and morphology of the wild accessions may play important roles in antibiosis effects. This germplasm offers a good resource for potato breeding to develop ZC resistance cultivars (Mora et al.). Wu et al. discovered and evaluated a thornless pummelo (*Citrus maxim*a var. “Mato Buntan”) bud-sport that grew more vigorously and was more tolerant to HLB than the thorny wild type. Further analysis indicated that the phenotype alterations of the bud-sport were associated with significant transcriptome changes, providing useful clues and targets for genetic breeding and gene editing for citrus improvement.

Current management strategies for HLB and ZC mainly rely on chemical control and integrated pest management measures to limit the spread of the pathogen. Recent developments in potato genetic resources and solanaceous crop improvement technologies are reviewed by Mora et al. These could be further leveraged for developing new potato cultivars with genetic resistance to the psyllid and *C*Lso. Yang et al. summarize the main aspects contributing to understanding *C*Las pathogenesis, including virulence targets, citrus defenses, and interactions with the host microbiome. They discuss the possible use of antimicrobial agents, plant defense activators, and beneficial bacteria as potential strategies to combat HLB.

## Final comment

In conclusion, the Research Topic articles cover a broad spectrum of *C*Las/*C*Lso-hosts interactions and may help to develop novel control strategies against HLB/ZC.

## Author contributions

XW drafted and prepared the final version of the editorial. KM, LP, MC, and CZ provided feedback and edits. All authors approved the editorial.

## Funding

This study was supported in part by funds from the National Key Research and Development Program of China (2021YFD1400800 and 2018YFD0201500) to XW and CZ. USDA-NIFA (2021-70029-36056), Texas A&M AgriLife Research Insect-vectored Disease Seed Grants (124185-96210), the Texas A&M AgriLife Institute for Advancing Health Through Agriculture to KM, from Fundecitrus, Grant No. 817526 from the European Union H2020 Innovation Action Program and Project PID2019-104569RB-I00 from the AEI-Spain to LP.

## Conflict of interest

The authors declare that the research was conducted in the absence of any commercial or financial relationships that could be construed as a potential conflict of interest.

## Publisher's note

All claims expressed in this article are solely those of the authors and do not necessarily represent those of their affiliated organizations, or those of the publisher, the editors and the reviewers. Any product that may be evaluated in this article, or claim that may be made by its manufacturer, is not guaranteed or endorsed by the publisher.

